# Adoption of a Digital Patient Health Passport as Part of a Primary Healthcare Service Delivery: Systematic Review

**DOI:** 10.3390/jpm12111814

**Published:** 2022-11-01

**Authors:** Tuan Yuswana Tuan Soh, Nik Mohd Mazuan Nik Mohd Rosdy, Mohd Yusmiaidil Putera Mohd Yusof, Syathirah Hanim Azhar Hilmy, Budi Aslinie Md Sabri

**Affiliations:** 1 Centre of Population Oral Health and Clinical Prevention, Faculty of Dentistry, Universiti Teknologi MARA, Sungai Buloh 47000, Selangor, Malaysia; 2 Centre of Oral and Maxillofacial Diagnostics & Medicine Studies, Faculty of Dentistry, Universiti Teknologi MARA, Sungai Buloh 47000, Selangor, Malaysia; 3 Institute of Pathology, Laboratory and Forensic Medicine (I-PPerForM), Universiti Teknologi MARA, Sungai Buloh 47000, Selangor, Malaysia

**Keywords:** health information technology, mobile health, consumer health informatics, healthcare technology personal digital assistant, personal identification systems

## Abstract

The utilization of digital personal health records is considered to be appropriate for present-time usage; it is expected to further enhance primary care’s quality-of-service delivery. Despite numerous studies conducted on digital personal health records, efforts in a systematic evaluation of the topic have failed to establish the specific benefits gained by patients, health providers, and healthcare systems. This study aimed to conduct a systematic review regarding the impact of digital personal health records in relation to the delivery of primary care. The review methods included five methodological elements that were directed by the review protocol 2020 (PRISMA). Over a time period of 10 years (2011–2021), 2492 articles were retrieved from various established databases, including Scopus, Web of Science, PubMed, EBSCO—Medline, and Google Scholar, and based on reference mining. The Mixed Method Appraisal Tool (MMAT) was used for quality appraisal. A thematic analysis was performed to develop the themes in this study. The thematic analysis performed on 13 articles resulted in seven main themes, which were empowering the patient, helping with communication, improving relationships, improving the quality of care, maintaining health records, sharing records, and saving time. We concluded the study by expanding the seven themes into 26 sub-themes, of which each served as answers to our main research question that prompted this systematic review.

## 1. Introduction

Healthcare systems are becoming increasingly complex; larger groups of diverse healthcare professionals are collectively working together over vast geographical distances for the purpose of optimal patient care delivery. These developments have inevitably cultivated international interest in information technology’s (IT) potential to simultaneously facilitate efficiency and reduce errors. [1]. The underlying assumption of introducing IT in healthcare is that efficient information flow will ultimately result in improved quality of care [2]. As the electronic medical record (EMR) system has been proven to play a significant role in improving healthcare services, full coverage of EMR for entire healthcare facilities has become the vision of many countries [3,4]. Consistent with the extensive use of Information and Communication Technology (ICT), which aims to improve the quality of health services delivery [5], healthcare providers also make up the top three primary users and contributors to big data in their daily practice [6,7]. The big data contribution can also be used by healthcare providers as a resource of micro-level socio-demographic information in enhancing patient care through a holistic approach. Apart from that, it can also be used by the national healthcare sector in monitoring the country’s burden of disease at the macro level. A health passport or a personalized health record is a tool that provides personalized health information either via mobile devices or any document that carries health record information [8,9,10].

The importance of personal health records in continuous care [11] and advances in technology, as well as patient capabilities [8], have led to the use of mobile applications, turning health passports into personalized digital records. The use of this personalized digital record has benefited both parties in improving communication and service delivery [12]. Readily available mobile applications such as passports are currently limited to specific functions in relation to a narrow spectrum of diseases that have fixed purposes, such as disease prevention and/or self-treatment directed towards patients with diabetes, obesity, or cancer [13,14]. The trend of mobile application utilization is predicted to rise exponentially with the simultaneous increase in interest in health-oriented applications and the vast majority of people owning smartphones [15]. According to Phelan (2020), the WHO recommended the use of a health identification passport to control the spread of Coronavirus 2 severe acute respiratory syndrome (SARS-CoV-2). The use of this mobile health passport is considered appropriate for present-day usage in ensuring effective communication to improve the quality-of-service delivery and warrant the continuity of care while navigating the strict physical distance measures imposed to control the current pandemic.

The delivery of information and sharing of health information can increase the awareness of health within the community. Ownership of personal health records has proven able to sustain the delivery of service. The role of the patient as an active partner in healthcare, and not just a passive object of diagnostic testing and medical treatment, is widely accepted [16,17]. Providing personal health information to patients is considered a crucial issue and the central focus of patient educational activities [18]. Global development shows technology capable of reducing access to information as a whole. The demand for health apps has rapidly increased as a result of improvements in mobile software and hardware as well as the availability of more linked devices. According to the most recent figures, there are currently more than 259,000 mHealth applications in app stores, with 3.2 billion downloads annually [19]. Healthcare organizations are dubious about the potential role that applications could play in healthcare because the clinical data supporting their usage independently of other therapies is still relatively weak. In the field of health, it has proven to be effective and produces significant results because, with a variety of visual methods in the form of images and videos, they can attract patients. However, the sharing of individual personal information is still debated. The lack of review and research is discussed related to the use of digital personal records among patients. From this background, the authors hope to determine the benefits of digital technology for patients who visit their primary health provider to solve a health problem. The purpose of this systematic review is to identify the benefits of the use of digital technology for personal health records for both patients and nurses.

## 2. Materials and Method

Several processes of systematic searching strategies, including identification, screening, and eligibility, were performed as means of ensuring a rigorous and methodical search.

### 2.1. Protocol and Registration

A systemic review was performed according to the guidelines of the Preferred Reporting item for Systematic Review and Meta-Analysis (PRISMA). PRISMA is an updated guideline for reporting systematic reviews and was published by [20] as an attempt to strengthen and maintain a sound methodology for developing a Systematic Review (SR) via increased transparency whilst simultaneously conserving the quality of the review. The method comprised five steps: (1) Literature review, (2) inclusion and exclusion criteria, (3) selection of studies, (4) quality appraisal, and (5) data extraction and synthesis. In addition, the rest of the contents of the Method section should be numbered and presented based on the mentioned steps. This systematic review was registered with the International Prospective Register of Systematic Review (PROSPERO) with ID 269,756 and registration number: CRD42021269756.

### 2.2. Research Question

The research questions were formulated by the authors by using the PICO mnemonic, which signifies ‘P’ (Population or Problem), ‘I’ (Intervention/interest), ‘C’ (Context), and ‘O’ (Outcome). Based on these concepts, the authors included four main aspects as part of the review, the provider and patient at primary care (Population), digital health passport (Intervention), primary care service (Context), and benefits (Output). PICO guided the authors to formulate the main research question of this study: How do users benefit from personal digital health records as part of primary care service?

### 2.3. Search Strategy

Based on the formulated research question, three main keywords were identified: Benefit, personal digital health records, and potential user. To enrich these keywords, the authors sought their synonyms, related terms, and variations by using APA, a thesaurus, and index terms to the keywords used by past studies, referring to the keywords suggested by Scopus and asking the opinion of experts. The list of keywords is tabulated in Appendix A. Based on this process, several MeSH term keywords similar to benefit, health passport, and potential user in primary care, including advantage, cost benefit, usage, acceptance, acceptable, acceptability, efficiency, success, useful, efficacy, use, effectiveness, helpful, value, usability, adaptation, adaptable, adaptability, Digital Personal Health Record, Digital Personal Health Information, Digital Personal Medical Record, Digital Personal Dental Record, Personal Health Record, Personal Health Information, Personal Medical Record, Personal Dental Record, Digital Mobile health app, Digital mobile dental app, Primary care User, Primary care Operator, Primary care Worker, Primary care Patient, Primary healthcare Operator, Primary healthcare Worker, Primary Healthcare Patient, Primary Healthcare provider, Healthcare provider, Healthcare professional, Primary Healthcare professional, Primary health care Operator, outpatient, Doctors, nurse, dental nurse, dental therapist, dental hygienist, dentist, medical assistance, nurse midwife, and health care provider, were checked. The combinations of these keywords were processed using search functions, such as field code functions, phrase searching, wildcards, truncation, and Boolean operators, in five databases: Scopus, Web of Science, PubMed, EBSCO—Medline, and Google Scholar. The detailed keyword search is presented in Appendix B. Based on the search, 2487 potential articles were identified from the selected databases. In addition, five articles were selected based on reference mining.

### 2.4. Identification

Identifying the selected papers was the second procedure carried out, where articles were either included or excluded (with the assistance of a references manager or manually screened by the authors) from the study based on a specific set of criteria, such as excluding duplicate articles from different databases. Checking was performed thoroughly in which, all articles with similarities in their title, year of publication, or author were considered duplicates. The study was performed employing a search and review of high-quality articles to maintain empirical data. Considering the concept of ‘research field maturity’ emphasized by [21], this review limited the screening process to only include articles published after the year 2000. Research field maturity is a framework that refines the scope of previous studies to complement the current trends in the resulting review and provide views on the maturity of the field to obtain a deeper understanding of the research area.

This timeline was chosen given that the number of published studies was sufficient to perform a representative review. The authors decided to review empirical research papers since they offer primary data. Notably, to avoid confusion, only those written in English were considered. This study focused only on research in the primary care setting. The authors manually checked the remaining papers (either by reading the title, abstract, or the entire paper) to identify whether the papers matched the established inclusion criteria. A total of 2433 articles were excluded from the review during this stage since they did not satisfy the inclusion criteria. The screening was performed by choosing the criteria for article selection, which was automatically performed based on the sorting function in the database. The screening process continued with sorting, using EndNote^TM^ 20 (Version 20.3—Bld 16073) as the database manager. This resulted in 54 remaining articles for examination in the next screening stage.

### 2.5. Screening

#### 2.5.1. Study Selection and Extraction

**Figure 1:**
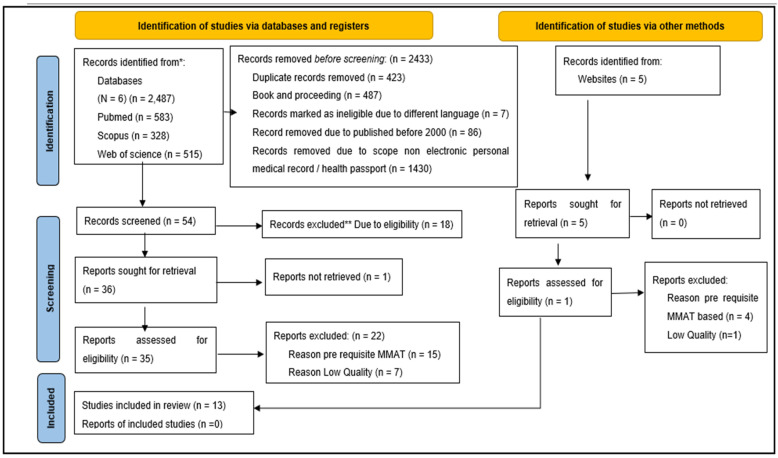
PRISMA 2020 flow diagram for new systematic reviews, which included searches of databases, registers, and other sources. * Consider, if feasible to do so, reporting the number of records identified from each database or register searched (rather than the total number across all databases/registers). ** If automation tools were used, indicate how many records were excluded by a human and how many were excluded by automation tools.

This systematic review utilized the mixed-methods appraisal tool (MMAT), version 2018, for the assessment of evidence quality that was incorporated into the study [22]. This tool has the ability to appraise the quality of empirical studies, including primary research based on experimentation, observation, and/or simulation in three separate categories of study: Qualitative, quantitative, and mixed methods. The selection and extraction process of articles is presented in the flowchart in Figure 1 (PRISMA 2020 flow diagram for new systematic reviews, which included searching databases, registers, and other sources), introduced by PRISMA, as mentioned earlier [23].

#### 2.5.2. Eligibility Criteria

This review systematically analyzed the articles whilst establishing the optimal method of delivering the integration of differences through the implementation of qualitative and quantitative synthesis to accommodate the study’s inclusion of various research designs. The assessment of study details was initiated by screening all papers with two questions: S1. ‘Are there clear research questions?’ and S2. ‘Do the collected data allow one to address the research questions?’ The assessment was allowed to proceed only when both screening questions were answered with a “yes”; paper retrieval was actively attempted by authors at this stage. The utilization of MMAT aided the authors by serving as guidance during the process of scrutinizing and selecting articles during screening; the chosen articles were included in the next step. All 54 remaining articles went through the 3rd phase of screening, which resulted in 19 articles being excluded: 18 due to eligibility, while one was due to the inability to retrieve the article. A manual search based on similar keywords on alternative databases resulted in the inclusion of an additional 5 articles, of which only 1 remained after the screening of eligibility of criteria for the next examination. Therefore, a total of 36 articles were retrieved at the initial stage of the systematic review, all of which needed to undergo further quality assessment.

#### 2.5.3. Assessment of Quality

The remaining articles were presented to two evaluators for quality assessment as a means of ensuring the articles’ quality in terms of their respective content. In this review, both evaluators agreed to use MMAT 2018 as a tool to rank the articles into 3 quality distinct categories, namely high (if the answer to more than 3 questions is ‘YES’), moderate (if the answer to 3 questions is ‘YES’), and low (if the answer is ‘YES’ to less than 3 questions), as suggested by Petticrew and Roberts [24]. The experts assessed quality by answering the questions in MMAT as proposed in the guideline. All aspects of the assessment were more focused on methodology. Sets of questions based on methodology are presented in Appendix C. The articles were exclusively included in this review only after a concurring agreement on their content quality by both experts at a minimally moderate level. The disagreement was discussed between them before deciding on the inclusion or exclusion of the articles for this review. This process ranked 11 articles as high quality, and 2 as moderate quality. Thus, all remaining articles were eligible for review.

### 2.6. Data Extraction and Data Analysis

An integrated review was used for this analysis. Diverse study designs (quantitative, qualitative, and mixed methods) were able to be included in the review because of this strategy. Utilizing qualitative or mixed-method techniques that aid in iterative comparison across the primary data source is the best way to analyze integrative data. The researcher carefully reviewed all 13 articles, paying particular attention to the abstract, findings, and Discussion sections. Any information from the examined studies that could help answer the research question was extracted and put in a table as part of the data extraction process, which was based on the research question.

The researcher then conducted a thematic analysis to find themes and sub-themes based on her efforts to identify patterns and topics in the extracted data by clustering, numbering, noting similarities, and marking relationships [25]. Thematic analysis is thought to be the best method for integrating a mixed research design [26]. It is described as a descriptive approach that condenses the data in a versatile manner and combines other data analysis methods [27]. It was initiated by the researchers familiarizing themselves with the dataset via active and thorough reading. This method was followed by the process of generating initial codes. NVIVO ver.11 (Release 1.6.1) was used to organize data at the granular level and categorize them into themes and subthemes. All the data related to the main research questions were selected and coded. This review practiced inductive coding where the themes were derived from the coded data. Inductive coding is the process of developing themes that have an association and reflect the whole subtheme and dataset under that theme [25]. The final process is a procedure of checking the developed themes for the analysis. The authors agreed to maintain seven themes and 26 sub-themes in the final analysis of this review.

## 3. Results

### 3.1. Study Selection

A total of 13 articles have been included to be further studied and analyzed [28,29,30,31,32,33,34,35,36,37,38,39,40]. Detailed demographics for all selected articles are presented in Table 1. During the quality assessment, the first examiner regarded nine articles to be of good quality, eight to be of moderate level, and four to be of poor quality. The second examiner ranked five items as being of excellent quality, eleven as being of medium level, and five as being of poor quality. Both examiners reached a consensus to include articles of medium and high quality for the subsequent assessment. Therefore, thirteen publications were selected for further analysis.

From the 13 articles, a total of seven papers focused their study on the United States of America (USA), [28,30,32,35,36,37,40], whereas [31] only one paper was from Germany [29], only one was from Japan [33], one was from Spain [34], and only one was from Australia. Lastly, two papers were from Canada [38,39].

It was documented that one article was a randomized controlled trial [35], four articles focused on quantitative analysis as a methodology [28,29,30,31], and the other six articles focused on qualitative methodology [32,33,36,37,39,40]. Two studies utilized a mixed-method approach [34,38].

Regarding the year of publication, 2020 produced the highest number of articles in which three studies [32,37,38] were published in that year and included in this review. This was followed by 2014 [35,36] and 2017 [34,40] in which two studies were published, respectively. No articles were found dated before 2011, while only one published article was included for all other years.

### 3.2. Developed Themes

The thematic analysis completed on 13 articles resulted in seven main themes, which include empowering patients, helping with communication, improving relationships, improving the quality of care, maintaining health records, sharing records, and saving time as elaborated in Table 2. To explore the seven themes, we expanded them into 26 sub-themes, which provided answers to our main research question for this systematic review: ‘How do users benefit from personal digital health records as part of primary care service?’.

### 3.3. Empowerment

The first sub-theme revolved around cultivating awareness among patients regarding their personal state of health. Tawara, Yonemochi [29], Hanna, Gill [34], and Davis and MacKay [38] agree that sharing health records with the patient provides awareness to the holder. Awareness is important to the patient and is able to empower patients in self-care [28,34]. The utilization of digital personal health records enables healthcare providers to disclose each patient’s specified health information on their current health status at any given time [32,36,37,38,39,40]. A systematic and well-kept record of each patient’s personal medical data is vital in sustaining the delivery of optimal healthcare service and improving patient health literacy [32,35].

### 3.4. Help in Communication

A total of four sub-themes were derived from this theme: Improving doctor–patient communication, improving communication between providers, improving the consultation behavior of the parent or patient, and receiving alert notification from the provider. By being able to readily assess their individual personal health records, patients will have an enhanced understanding of their own health status, which will propel effective patient–doctor communication [33,34,37,39,40]. Furthermore, a personally controlled electronic health record can help specialists and general practitioners communicate when managing their patients [31,34,37,39]. Interactive communication and active participation will help both parties achieve mutual understanding. Communication between the parent or patient and provider or communication between providers will improve patient management as a whole and facilitate seamless care [31,39]. Ruiz and Andrade [30] and Lopez Segui and Pratdepadua Bufill [33] mentioned that using digital platforms is beneficial in providing alert messages from the provider to the patient based on the received data. However, Aston and Sweet [39] stated that not all healthcare information is relevant enough to be shared with patients, and other departments should avoid misinterpreting the concept.

### 3.5. Improve Relationship

This theme discussed doctor–patient relationships as well as doctor–doctor relationships as a sub-theme. Digital personal health records will not only enhance the quality of the relationship between healthcare providers and patients but will also create a convenient environment for both parties during treatment sessions [28,40]. Previous authors [33] produced results that indicated a digital platform for personal health records will aid in the achievement of establishing an integration model in doctor–patient relationships.

### 3.6. Improve Quality of Care

This theme produced a total of eight subthemes: Improving the quality of care, supporting patient management, supporting decision making during the consultation, improving the type of treatment with prevention, patient monitoring, improving health status including oral health, improving child behavior, and lastly, the continuity of care. The usage of digital personal health records will provide an updated version of health information for both the patient and provider, which is inevitably expected to improve the quality of care [28,39]. For example, ‘*a study done in US reported that personal health records were able to improve patient care in hypertension*’ [35]. According to Abelson and Kaufman [40], ‘*Digital personal health records can also serve as an intervention in preventing further complications’*. The digital platform provides great assistance to patients in managing their health visits, seeking care, and updating their personal health information [36,38]. Sharing trustworthy and updated health data will help patients develop more of an understanding of their health and will help their assigned healthcare providers during the consultation and enhance the effectiveness of decision making [33,34,38,39]. Acknowledging the importance of disseminating personal health information in order to support patient management, Aston and Sweet [39] provided an opinion about diagnosis, in which the patient should be treated as dynamic and diagnosis should be personal to the patient and their family to avoid misunderstandings and create an uneventful treatment session. Increasing patient health knowledge by using digital personal health records will improve the treatment regarding prevention [31,35,40] by minimizing follow-up visits [40], increasing preventive management compared to active treatment [35], or the improvement of health status including oral health [31]. This digital record will help patients with self-monitoring [40]. ‘*Personally Control Electronic Health Record (PCEHR) help to improve patient healthcare*’ [34]. Furthermore, digital personal health records will help providers to manage children’s behavior via early awareness of challenges and any difficult behavior [39] through less curative treatment and improvements in the type of treatment regarding prevention [31]. The implementation of digital health records will create continuity of care through the means of enhanced communication [34] and sharing of records, especially in transient care between pediatric and adult healthcare [37,39].

### 3.7. Maintaining Health Records

Five sub-themes were identified under this theme: Access to health records, patients owning their medical records, improving health record documentation, documenting the state of health, and lastly, improving record accuracy. Digital personal health records will aid the service in terms of the provision of care with the availability of quality and trustworthiness of personal health data [33,38,39], especially for the older generation with physical and mental comorbidities [30]. By sharing health information on a digital platform, the patient will take responsibility and ownership [39]. Ownership and authorship of health records will improve the accuracy of documentation while also keeping it updated [28,31].

### 3.8. Records Sharing

Two sub-themes were identified in the records-sharing themes: Sharing health records with providers and sharing health records with family members. Sharing health records with providers either at the primary care level or in the specialist department is a great achievement when patients use the digital platform [29,32,33,36]. Sharing data with providers serves as a crucial component, especially during first-time visits and for patients with complex healthcare needs, in which the trustworthiness of the data needs to be maintained in order to create seamless care [34]. A study performed in the United States stated that 88% (*n* = 15) of users in a pilot study used the digital platform and were able to share their information with all providers across various departments such as their primary pediatrician, school nurse, dentist, medical specialist, and other emergency rooms [37]. Similarly, regarding sharing health records with family members, digital health records have the ability to share important messages with family members for emergency use [29], and can also serve as a medium to communicate the individual’s health issues to support patient management [39].

### 3.9. Time Saving

The time-saving theme involved saving time while using digital personal health records. This theme also discussed aspects of digital personal health records in relation to the element of time saving. [28]. Digital personal health records also provide patients with knowledge; this fact enables patients to be self-sufficient and conduct self-monitoring, which could save them time that would be potentially lost when seeking extra help associated with the severe state of their existing disease and its complications [40].

## 4. Discussion

This review aims to identify the benefits of digital personal health records by answering several research questions. As can be seen from the 13 publications, all of the research findings were positive, indicating that users had a favorable opinion of digital personal health records. Out of the thirteen articles, two studies focus on exploring the difficulties of using digital personal health records [36,40]. Only a few barriers, involving data flow and data security, have been discovered by researchers. The findings indicate that using digital personal health records can provide a variety of advantages, such as improving health-related communication. Furthermore, customers believe that by utilizing this technology, their access to healthcare and subsequent health outcomes would improve [40]. However, a study conducted by Donovan (2020) supported the hypothesis that using digital personal health records will increase communication and ease some of the difficulties in service delivery [37].

Digital personal health records readily offer immense benefits to potential users in numerous ways; ensuring continuous care by means of keeping nurses connected as well as enhancing communication between key stakeholders of member care plans [41,42]. Medical history information that has been made accessible warrants trained and authorized providers to resume the appropriate treatment plan according to the patient’s problems. Medical errors as well as inappropriate medication prescriptions involving potential adverse reactions, drug interactions, and worsening of chronic health problems are simultaneously prevented with the usage of patient health records [43].

Empowerment was the most frequently reflected theme in users’ perceptions, which was reported in ten articles in this review. Empowering patients is the most important element in the new paradigm of healthcare delivery to make sure the treatment delivered by doctors ultimately yielded positive results [44]. By knowing their personal health status and receiving updated health information, patients are directly made aware and propelled to actively control their existing health conditions, which will enhance the effectiveness of consultations and maintain their quality of life [45]. The application of the digital health record will provide patients with updated information on various diseases, risk factors associated with different diseases, signs and symptoms that manifest in varying diseases, and methods of early detection, as well as the management and treatment specifically targeted toward specified diseases, all of which is meant to inculcate a vast depth of knowledge as well as immense comprehension in health literacy for all patients [46,47,48,49].

Of the past ten studies, five studies used qualitative methods, two used quantitative methods, two used a mixed-method approach, and one used a randomized controlled trial (RCT). All previous studies reported positive outcomes on the use of digital personal health records to increase the patient’s knowledge and empower patients to engage in health and management decisions. This has been proven by a study conducted by [35] using RCT techniques. RCT was reported as the gold standard since it provided the highest degree of evidence and have the capacity to prevent all types of bias [50].

Studies related to personal health records in the delivery of primary care services varied from the use of appropriately sized documents to be carried and used as patient identification [51]. Out of the eight previous articles, five studies used a qualitative method, two studies used quantitative methods, and one study used mixed methods. All opinions showed that the use of digital personal health records can help communication related to health between patients and providers as well as among providers. However, there is one study that used qualitative methods, which reported that only specific information is appropriate to share with patients and should be verified by providers to prevent confusion. However, we know that this qualitative study only used respondents’ words based on local situations and cultured analysis; they did not show general results [52]. However, this theme is valid as it was also a result of two studies that were using mixed methods and two quantitative studies, and studies that used mixed methods were higher in value compared to other studies [53]. Without prejudice to any studies, the suggestions given by other studies, such as quantitative studies, should be taken into account. For example, it would still be feasible to facilitate communication between patients and providers and offer real access to classified patient information.

The role of the patient as an active partner in healthcare and not just a passive object of diagnostic testing and medical treatment is widely accepted [16,17]. The results of this review show that four previous studies have supported the use of digital personal health records to improve health relationships between patients and providers as well as among providers, three of which conducted qualitative studies while 1 was quantitative. Of the three previous studies, only one study mentioned taking steps to prevent bias [54]. While two studies showed that using random sampling is a step to avoid the risk of bias, it was not specifically stated. Furthermore, the quantitative study used a questionnaire that required self-responses, which exposed the study to reporting bias. However, this review agreed that the theme remained valid and was accepted, even though only one study used quantitative studies as a study design, as quantitative studies are superior to qualitative and both studies outlined the same theme [55].

The use of this digital personal health records application has benefited both parties in improving service delivery [56]. At the same time, there are ten previous studies analyzed in this review reporting the same result. Based on the perception of the users, studies found that using personal health records based on a digital platform can improve the quality of patient management. Half of the studies used a qualitative research design, while two were quantitative studies, two used a mixed-methods approach, and one was an RCT. This theme was valid as all of the past studies in this review, with their various research designs, reported the same results. The studies that used an RCT design confirmed the results in other studies [50], while the qualitative and quantitative studies were complimentary of each other [55] and the two studies that used a mixed-methods approach provided high report value [53].

Maintaining health records is an important element in healthcare delivery to sustain patient management and improve the quality of healthcare service [57]. For reports on the theme of maintaining health records, there were three studies that used quantitative methods and two studies using qualitative methods, while one study used a mixed-methods technique. For quantitative studies, all studies used random samples, various scales were used and were appropriate for the selected population, and all reports are well-tabulated. However, the result was recorded based on self-reports by the respondents. This shows that there is reporting bias as all self-administered measures are highly dependent on the honesty and understanding of the participants in answering the questions. The qualitative method only reports the words of the respondent based on the answers to the open-ended question, and the reported results focus on exploring the ideas more than the cultural analysis. All six previous studies used the cross-sectional study design, which showed respondent perception reports at certain points in time. The environment can change the result to be a negative perception of the respondent [55]. Therefore, reports on the use of personal health records using digital platforms to maintain health records need to be studied in detail using proper research techniques and standard measurement tools.

Information sharing is also a positive perception reported by seven articles in this research. Sharing the personal information of a patient is important for both providers and the patient [58]. This set of information is required at different times, for example, for emergency treatment sessions, medical consultations, laboratory tests, and hospital admission. This perception can change according to the current environment as these studies are cross-sectional studies and show that respondents have a positive perception of digital personal health records. The results of a cross-sectional study are measured at one point, and there is a possibility of obtaining different results when measured at different time points [59]. However, in this study, the results show that there is no clear difference between what health professionals reported and what patients reported. This shows that sharing information is important for both patients and providers. However, no clear measurements have been reported as most of these previous studies used qualitative methods and recorded the words of selected respondents. Respondents’ answers based on open-ended questions were recorded and shown in past studies through a qualitative design based on the suggested guidelines [60]. However, only one article was a quantitative study and one used a mixed-method approach, using convenient sampling in a small sample of outpatients. Therefore, the results of these studies do not indicate the real population ratio [61]. However, this result can be used to educate new users about the implementation of digital personal health records.

Time saving is one of the themes that was less reported in previous studies in this review. Only two studies provided a report on saving time, one quantitative and one qualitative method. Both studies are in accordance with their respective research guidelines with appropriate sampling. Thus, the results of their research can be reflected in the population. However, this study is cross-sectional and shows the results at a certain time, and it may change according to the environment, such as the stability of the system that uses digital platforms and internet access. However, there is no standard scale used to measure the extent to which it saves time. Further studies should be conducted with standard measurements to measure the extent of time saving while using a digital personal health record.

According to this systematic review, several research gaps have been identified. First, there is a need to understand the challenges and barriers encountered when using digital personal health records, even though individuals are aware of the high impact of using and sharing health data on their quality of life. Second, there is a need to obtain a good understanding of the data flow in the digital platform to ensure the data that patients share with the provider and their families is correct and reliable data. In addition, although previous studies have emphasized the benefits of digital health records, there is a need to understand the benefits, cost-effectiveness, challenges, and barriers at the administration level and for policymakers, not only from the user’s perspective but also the impact on the health system.

Existing gaps can be reduced if future scholars can focus on implementing a bigger scope, consider quantitative, qualitative, and mixed-methods approaches, produce more systematic integrative studies, and develop more publication standards. The empirical data of this study illustrates that the design of qualitative, quantitative, and mixed-method research focuses on existing literature on the importance of the use of digital personal health records. All approaches have different advantages and therefore should be investigated by future scholars with a bigger scope. The qualitative design offers a good and comprehensive source of explanation of processes in identified local contexts, driving empirical data to produce richer data that go beyond quantitative statistics. On the other hand, the mixed-method approach can improve data validity, establish a collection of second data sources, facilitate the creation of knowledge, and simultaneously integrate components that provide deeper and more detailed discovery toward obtaining a concrete conclusion.

Overall, the findings from previous studies outline the benefits of using digital personal health records in improving the quality-of-service delivery to patients [62]. Digital personal health records help reduce miscommunication between patients and nurses and help other nurses provide continuity of care with information sharing. These findings also emphasize that the development of health passports needs to be planned systematically to ensure their use and ensure consumers receive optimal benefits [41]. Empowering people in maintaining health is an important element of life continuity and well-being. Therefore, we suggest future studies explore how the usage of digital personal health records will have a positive impact and increase health outcomes.

There are some limitations in this study. Firstly, although Gusenbauer and Haddaway [63] suggested 14 potential databases to find relevant articles, due to limitations in access to these databases, only five databases were used, namely Scopus, Web of Science, PubMed, EBSCO—Medline, and Google Scholar. Secondly, the assessment of the quality process depends on the Mixed Method Appraisal Tool (MMAT). It is expected that the article will highlight quality variations if the checking is based on different quality assessment tools. However, Shaffril and Samah [64] emphasized that quality assessment is not solely intended to find the perfect article but rather find articles that fit the purpose of the review. Thirdly, despite conducting a systematic review, it is also encouraged to look objectively or perform a meta-analysis due to the benefits of better estimating the population as a whole rather than in a single study, such as its capacity to reduce tendencies given the heterogeneity of the methodologies employed in chosen studies.

## 5. Conclusions

In conclusion, having a digital personal health record resulted in significantly improved quality of medical care and increased use of medical services among patients. Personal health records could provide a relatively low-cost scalable strategy for improving the medical care for patients with comorbid medical and serious mental illnesses. It is beneficial if primary care is able to make an investment by incorporating digital personal health records into primary care; digital personal health records should also be made available and accessible to the entire community.

This paper systematically reviewed previous studies related to the use of a digital platform for personal health records in the setting of primary healthcare. This study used a systematic approach, and 13 articles were appraised for the scope of study and quality. Since the evaluation relied on the diversity of study designs, a thematic analysis of the 13 articles that were chosen produced seven key themes: (1) Empowering the patient, (2) helping with communication, (3) improving relationships, (4) improving the quality of care, (5) maintaining health records, (6) record sharing, and (7) time saving. As a conclusion of these seven themes, we expanded the main themes into 26 sub-themes, all of which aimed at providing answers to our main research question for this systematic review.

The review concluded that sharing personal health records via a digital platform in the delivery of health in the primary care setting is beneficial for all parties in maintaining the quality of care and improving the healthcare delivery system. Sharing personal health records prior to visits with providers will help by prompting both parties to engage in interactive communication and active participation in decision making, which will save time for the providers and for the patients themselves. Aside from saving time, personal health records are also able to improve doctor–patient communication and doctor–patient relationships. Strong social relationships will give each party the same level of purpose and responsibility. This will encourage each party, whether patient and provider or between providers, to help each other in patient management. Therefore, we can conclude that the usage of digital personal health records will create seamless care and lead to continuity of care.

## Figures and Tables

**Table 1:** Demographics of the study.

No	Title	Author	Type of Study	Country	Sample	Type of Sample
1	Many Physicians Are Willing to Use Patients’ Electronic Personal Health Records, But Doctors Differ by Location, Gender, And Practice	[28]	Cross sectional	United States	physicians—856 responds	random-sample
2	Use of patients’ mobile phones to store and share personal health information: results of a questionnaire survey	[29]	A cross-sectional questionnaire survey	Japan	193 patients	Convenience sampling—consecutive outpatients who visited our weekday clinic between 1 March and 30 May 2012
3	The Association of Graph Literacy with Use of and Skills Using an Online Personal Health Record in Outpatient Veterans	[30]	cross-sectional survey	United States	600 veterans We conducted a cross-sectional survey of veterans receiving outpatient care	conveniently recruited
4	Dissemination and use of the children’s dental pass in Germany	[31]	Cross sectional self-administered mail questionnaire	Germany	1086 Dental officers	Convenient
5	User-centred design and enhancement of an electronic personal health record to support survivors of paediatric cancers	[32]	Focus groups and structured interviews	United States	Paediatric cancer (*n* = 3), parents (*n* = 11), and healthcare providers (*n* = 14)	purposive sampling
6	The Prescription of Mobile Apps by Primary Care Teams: A Pilot Project in Catalonia	[33]	focus groups	Spain (Catalonia)	32 doctors and 79 patients per professional	purposive sampling
7	Patient perspectives on a personally controlled electronic health record used in regional Australia: ‘I can be like my own doctor’	[34]	semi-structured telephone interviews	Australia	12 patients	Random All Medenotes registered patients (*n* = 154) were emailed once and invited to participate in a telephone interview.
8	Randomized trial of an electronic personal health record for patients with serious mental illnesses	[35]	Randomized trial	United States	170 individuals	with a serious mental disorder and a comorbid medical condition treated in a community mental health center
9	Understanding patient perceptions of the electronic personal health record	[36]	qualitative study	United States	21 adults reporting an average age of about 64 years	purposive sampling—Identified
10	Design and implementation of a patient passport in a pediatric cardiology clinic	[37]	prospective survey—Qualitative	United States (New York)	100 patients	A total of 100 patients were enrolled in the study between October 2016 and November 2018.
11	Moving Beyond the Rhetoric of Shared Decision-Making: Designing Personal Health Record Technology with Young Adults with Type 1 Diabetes	[38]	Cross sectional Mixed method	Canada	22 participants took part, comprising 7 young adults with T1D and 15 care providers.	The two study groups were patients (young adults with T1D, aged 18–24 years) and healthcare providers of the patient population (specialist dieticians and nurses).
12	Snap shot: Achieving better care through a one-page personal health profile	[39]	qualitative descriptive approach	Canada	13 participants.	Participant self-identify as having a child with an ID who required additional support
13	Barriers and benefits to using mobile health technology after operation: A qualitative study	[40]	Qualitative	New York	800 participants and 25 individuals	800 participants from national surveys—randomly 25 individuals—telephones with 2 open ended question by phone.

**Table 2:** Analysis results for benefits of using digital personal health records.

Theme (Benefit)	Sub-Themes	Articles
Empower patient	Awareness to patient about their health	[29,34,38]
	Improve health literacy	[32,35]
	Empower patient to selfcare	[28,34]
	Improve knowledge	[32,36,37,38,39,40]
Help in Communication	Improve doctor—patient Communication	[33,34,37,39,40]
	Improve Communication among providers	[31,34,37,39]
	Improve consultation behaviour of parent/patient	[31,39]
	Receive alert notification from provider	[30,33]
Improve Relationship	Improve Dr-Patient relationship	[28,33,39,40]
Improve quality of care	Improve quality of care	[28,34,35,39,40]
	Support patient management	[36,38,39]
	Support Decision making during consultation	[33,34,38,39],
	Improve type of treatment with prevention	[31,35,40]
	Patient monitoring	[40]
	Improve Health status	[34,35,40]
	Improve oral health status	[31]
	Improve children behaviour	[31,39]
	Continuity of care	[37,39]
Maintain Health Records	Patient own medical records	[39]
	Improve record documentation	[28]
	Improve records accuracy	[28]
	Document health state	[31]
	Accessing health records	[30,33,38,39]
Records sharing	Sharing Health record with providers	[29,32,33,34,36,37]
	Sharing health record with family	[29,39]
Time saving	Save time to see doctor	[28,40]

## Appendix A. Searching Systematic Review

Question based on PICO concept

Population/problem/patient	Intervention	Comparison/context	Outcome
Primary care User	Using the Digital health passport	Not using the Digital health passport	Benefit

How useful the Digital health passport to primary care user?

Keyword

1. Synonym
2. Related term
3. Variation

	Population/problem/patient	Intervention	Comparison	Outcome
PICO	Primary care User	Using the Digital health passport	Not using the mobile health app	Benefit
	Primary care Operator	Digital Personal Health Record(s)		Acceptable
	Primary care Worker	Digital Personal Health Information(s),		Efficiency
	Primary care Patient	Digital Personal Medical Record(s)		Success
	Primary healthcare Operator	Digital Personal Dental Record(s)		Usefulness
	Primary healthcare Worker	Personal Health Record(s)		Efficacy
	Primary Healthcare Patient(s)	Personal Health Information(s),		Use
	Primary Healthcare providers	Personal Medical Record(s)		Effectiveness
	Healthcare providers	Personal Dental Record(s)		Helpful
	Healthcare professional	Digital Mobile health app		Value
	Primary Healthcare professional	Digital mobile dental app		Usability
	Primary health care Operator	Digital health passport usage		Inefficient
	Patient(s)			Ineffective
	Doctor(s)			useless
	Nurse(s)			Advantage
	dental therapies			
	dental hygienist(s)			
	midwife			

Platform Searching

I. PubMed
II. Scopus
III. EBSCO
IV. Web of Science
V. Google Scholar

## Appendix B. Searching Strategy Result

I. PubMed:

**No.**	**Concept**	**Search String: PubMed**	**Number**
#1	Benefit/Advantage	((((((((((((((((((advantage s[MeSH Terms]) OR (analysis, cost benefit[MeSH Terms])) OR (analysis, cost benefit[MeSH Terms])) OR (usage[MeSH Terms])) OR (acceptance(s)[MeSH Terms])) OR (acceptable[MeSH Terms])) OR (acceptability[MeSH Terms])) OR (efficiency[MeSH Terms])) OR (success[MeSH Terms])) OR (usefull[MeSH Terms])) OR (efficacy[MeSH Terms])) OR (use[MeSH Terms])) OR (effectiveness[MeSH Terms])) OR (helpfull[MeSH Terms])) OR (value[MeSH Terms])) OR (usability[MeSH Terms])) OR (adaptation[MeSH Terms])) OR (adaptable[MeSH Terms])) OR (adaptability[MeSH Terms])	278,496
#2	Digital Health passport usage	(((((((((((Digital health passport[MeSH Terms]) OR (Digital Personal Health Record(s)[MeSH Terms])) OR (Digital Personal Health Information(s)[MeSH Terms])) OR (Digital Personal Medical Record(s)[MeSH Terms])) OR (Digital Personal Dental Record(s)[MeSH Terms])) OR (Personal Health Record(s)[MeSH Terms])) OR (Personal Health Information(s)[MeSH Terms])) OR (Personal Medical Record(s)[MeSH Terms])) OR (Personal Dental Record(s)[MeSH Terms])) OR (Digital Mobile health app[MeSH Terms])) OR (Digital mobile dental app[MeSH Terms])) OR (Digital health passport usage[MeSH Terms])	17,223
#3	Potential User	((((((((((((((((((((((Primary care User[MeSH Terms]) OR (Primary care Operator[MeSH Terms])) OR (Primary care Worker[MeSH Terms])) OR (Primary care Patient[MeSH Terms])) OR (Primary healthcare Operator[MeSH Terms])) OR (Primary healthcare Worker[MeSH Terms])) OR (Primary Healthcare Patient(s)[MeSH Terms])) OR (Primary Healthcare provider(s)[MeSH Terms])) OR (Healthcare provider(s)[MeSH Terms])) OR (Healthcare professional[MeSH Terms])) OR (Primary Healthcare professional(s)[MeSH Terms])) OR (Primary health care Operator(s)[MeSH Terms])) OR (out patients[MeSH Terms])) OR (Doctors[MeSH Terms])) OR (nurses[MeSH Terms])) OR (dental nurse(s)[MeSH Terms])) OR (dental therapist[MeSH Terms])) OR (dental hygienist[MeSH Terms])) OR (dental hygienists[MeSH Terms])) OR (medical assistance[MeSH Terms])) OR (nurse midwife[MeSH Terms])) OR (midwife[MeSH Terms])) OR (health care provider[MeSH Terms])	847,494
	Total	(#1 AND #2 AND #3)	**583**

II. SCOPUS:

**No.**	**Concept**	**Search String: SCOPUS**	**Number**
#1	Benefit	(advantage* OR cost benefit OR usage OR acceptance* OR acceptable OR acceptability OR efficiency OR success OR useful OR efficacy OR use OR effectiveness OR helpful OR value OR usability OR adaptation OR adaptable OR adaptability)	1,932,408
#2	Digital Health passport usage	(Digital health passport* OR Digital Personal Health Record* OR Digital Personal Health Information* OR Digital Personal Medical Record* OR Digital Personal Dental Record* OR Personal Health Record* OR Personal Health Information* OR Personal Medical Record* OR Personal Dental Record* OR Digital Mobile health app* OR Digital mobile dental app* OR Digital health passport usage)	3449
#3	Potential User	(“Primary care User*” OR “Primary care Operator*” OR “Primary care Worker” OR “Primary care Patient*” OR “Primary healthcare Operator*” OR “Primary healthcare Worker*” OR “Primary Healthcare Patient*” OR “Primary Healthcare provider*” OR “Healthcare provider*” OR “Healthcare professional” OR “Primary Healthcare professional*” OR “Primary health care Operator*” OR out*patient* OR Doctors OR nurse* OR “dental nurse*” OR “dental therapist*” OR “dental hygienist*” OR dentist* OR “medical assistance” OR “nurse midwife” OR midwife OR “health care provider*”)	1,314,396
	Total	(#1 AND #2 AND #3)	**328**

III. EBSCO:

**No.**	**Concept**	**Search String: EBSCO**	**Number**
#1	Benefit	advantage* OR “cost benefit” OR usage OR acceptance* OR acceptable OR acceptability OR efficiency OR success OR useful OR efficacy OR use OR effectiveness OR helpful OR value OR usability OR adaptation OR adaptable OR adaptability	6,951,959
#2	Digital Health passport usage	“Digital health passport*” OR “Digital Personal Health Record*” OR “Digital Personal Health Information*” OR “Digital Personal Medical Record*” OR “Digital Personal Dental Record*” OR “Personal Health Record*” OR “Personal Health Information*” OR “Personal Medical Record*” OR “Personal Dental Record*” OR “Digital Mobile health app*” OR “Digital mobile dental app*” OR “Digital health passport usage”	1381
#3	Potential User	“Primary care User*” OR “Primary care Operator*” OR “Primary care Worker*” OR “Primary care Patient*” OR “Primary healthcare Operator*” OR “Primary healthcare Worker*” OR “Primary Healthcare Patient*” OR “Primary Healthcare provider*” OR “Healthcare provider*” OR “Healthcare professional*” OR “Primary Healthcare professional*” OR “Primary health care Operator*” OR “out*patient*” OR doctor* OR nurse* OR “dental nurse*” OR “dental therapist*” OR “dental hygienist*” OR “dentist*” OR “medical assistance*” OR “nurse midwife” OR “midwife” OR “health care provider*”	588,313
	Total	(#1 AND #2 AND #3)	**231**

IV. Web of Sciences

**No.**	**Concept**	**Search String: Web of Sciences**	**Number**
#1	Benefit	(ALL = ((advantage* OR “cost benefit” OR usage OR acceptance* OR acceptable OR acceptability OR efficiency OR success OR useful OR efficacy OR use OR effectiveness OR helpful OR value OR usability OR adaptation OR adaptable OR adaptability)))	27,747,961
#2	Digital Health passport usage	ALL = ((“Digital health passport*” OR “Digital Personal Health Record*” OR “Digital Personal Health Information*” OR “Digital Personal Medical Record*” OR “Digital Personal Dental Record*” OR “Personal Health Record*” OR “Personal Health Information*” OR “Personal Medical Record*” OR “Personal Dental Record*” OR “Digital Mobile health app*” OR “Digital mobile dental app*” OR “Digital health passport usage”))	2633
#3	Potential User	ALL = ((“Primary care User*” OR “Primary care Operator*” OR “Primary care Worker*” OR “Primary care Patient*” OR “Primary healthcare Operator*” OR “Primary healthcare Worker*” OR “Primary Healthcare Patient*” OR “Primary Healthcare provider*” OR “Healthcare provider*” OR “Healthcare professional*” OR “Primary Healthcare professional*” OR “Primary health care Operator*” OR “out*patient*” OR doctor* OR nurse* OR “dental nurse*” OR “dental therapist*” OR “dental hygienist*” OR “dentist*” OR “medical assistance*” OR “nurse midwife” OR “midwife” OR “health care provider*”))	1,258,928
#4	Total	(#1 AND #2 AND #3)	515

V. Google Scholar

Step

1. Using engine
2. URL: https://harzing.com/resources/publish-or-perish (accessed on 1 August 2021)
3. Search String

advantage* OR “cost benefit” OR usage OR acceptance* OR acceptable OR acceptability OR efficiency OR success OR useful OR efficacy OR use OR effectiveness OR helpful OR value OR usability OR adaptation OR adaptable OR adaptability AND “Digital health passport*” OR “Digital Personal Health Record*” OR “Digital Personal Health Information*” OR “Digital Personal Medical Record*” OR “Digital Personal Dental Record*” OR “Personal Health Record*” OR “Personal Health Information*” OR “Personal Medical Record*” OR “Personal Dental Record*” OR “Digital Mobile health app*” OR “Digital mobile dental app*” OR “Digital health passport usage” AND “Primary care User*” OR “Primary care Operator*” OR “Primary care Worker*” OR “Primary care Patient*” OR “Primary healthcare Operator*” OR “Primary healthcare Worker*” OR “Primary Healthcare Patient*” OR “Primary Healthcare provider*” OR “Healthcare provider*” OR “Healthcare professional*” OR “Primary Healthcare professional*” OR “Primary health care Operator*” OR “out*patient*” OR doctor* OR nurse* OR “dental nurse*” OR “dental therapist*” OR “dental hygienist*” OR “dentist*” OR “medical assistance*” OR “nurse midwife” OR “midwife” OR “health care provider*”

4. Filter

Select the maximum searching 1000.

Uncheck Include citation and include patent

5. Save to endnote
6. Create a folder for Google Scholar

Result: 830

## Appendix C. The Criteria Used to Determine the Rigour of the Methodology and Analysis Used in the Selected Articles

**Table A1:** The Criteria Used to Determine the Rigour of the Methodology and Analysis Used in the Selected Articles.

Research Design	Assessment Criteria
Qualitative	QA1—Is the qualitative approach appropriate to answer the research question? QA2—Are the qualitative data collection methods adequate to address the research question? QA3—Are the findings adequately derived from the data? QA4—Is the interpretation of results sufficiently substantiated by data? QA5—Is there coherence between qualitative data sources, collection, analysis and interpretation?
Quantitative (descriptive)	QA1—Is the sampling strategy relevant to address the research question? QA2—Is the sample representative of the target population? QA3—Are the measurements appropriate? QA4—Is the risk of nonresponse bias low? QA5—Is the statistical analysis appropriate to answer the research question?
Quantitative (non-randomised)	QA1—Are the participants representative of the target population? QA2—Are measurements appropriate regarding both the outcome and intervention (or exposure)? QA3—Are there complete outcome data? QA4—Are the confounders accounted for in the design and analysis? QA5—During the study period, is the intervention administered (or exposure occurred) as intended?
Mixed methods	QA1—Is there an adequate rationale for using a mixed methods design to address the research question? QA2—Are the different components of the study effectively integrated to answer the research question? QA3—Are the outputs of the integration of qualitative and quantitative components adequately interpreted? QA4—Are divergences and inconsistencies between quantitative and qualitative results adequately addressed? QA5—Do the different components of the study adhere to the quality criteria of each tradition of the methods involved?

Source: Hong, Q.N.; Fàbregues, S.; Bartlett, G.; Boardman, F.; Cargo, M.; Dagenais, P.; Gagnon, M.-P.; Griffiths, F.; Nicolau, B.; O’Cathain, A. The Mixed Methods Appraisal Tool (MMAT) version 2018 for information professionals and researchers. *Educ. Inf.* **2018**, *34*, 285–291. Hong, Fàbregues et al. [22].
